# Switching from Beraprost to Selexipag in the Treatment of Pulmonary Arterial Hypertension: Insights from a Phase IV Study of the Japanese Registry (The EXCEL Study: EXChange from bEraprost to seLexipag Study)

**DOI:** 10.3390/ph17050555

**Published:** 2024-04-26

**Authors:** Yuichi Tamura, Hiraku Kumamaru, Ichizo Tsujino, Rika Suda, Kohtaro Abe, Takumi Inami, Koshin Horimoto, Shiro Adachi, Satoshi Yasuda, Fusako Sera, Yu Taniguchi, Masataka Kuwana, Koichiro Tatsumi

**Affiliations:** 1 Pulmonary Hypertension Center, International University of Health and Welfare Mita Hospital, Tokyo 108-8329, Japan; 2 Department of Healthcare Quality Assessment, Graduate School of Medicine, The University of Tokyo, Tokyo 113-8654, Japan; kumamaru-tky@umin.ac.jp; 3 Division of Respiratory and Cardiovascular Innovative Research, Faculty of Medicine, Hokkaido University, Sapporo 060-8638, Japan; itsujino@med.hokudai.ac.jp; 4 Department of Respirology, Graduate School of Medicine, Chiba University, Chiba 260-8670, Japan; sudarika@chiba-u.jp (R.S.); tatsumi@faculty.chiba-u.jp (K.T.); 5 Department of Cardiovascular Medicine, Faculty of Medical Sciences, Kyushu University, Fukuoka 812-8582, Japan; abe.kotaro.232@m.kyushu-u.ac.jp; 6 Department of Cardiovascular Medicine, Kyorin University School of Medicine, Tokyo 160-8582, Japan; tinami@ks.kyorin-u.ac.jp; 7 Department of Cardiovascular Medicine, Matsuyama Red Cross Hospital, Matsuyama 790-0826, Japan; 8 Department of Cardiology, Nagoya University Hospital, Nagoya 466-8560, Japan; sadachi@med.nagoya-u.ac.jp; 9 Department of Cardiovascular Medicine, Tohoku University Graduate School of Medicine, Sendai 980-0872, Japan; 10 Department of Cardiovascular Medicine, Osaka University Graduate School of Medicine, Suita 565-0871, Japan; 11 Division of Cardiovascular Medicine, Department of Internal Medicine, Kobe University Graduate School of Medicine, Kobe 650-0017, Japan; yu.taniguchi007@gmail.com; 12 Department of Allergy and Rheumatology, Nippon Medical School Graduate School of Medicine, Tokyo 113-8602, Japan; kuwanam@nms.ac.jp; 13 Scleroderma/Myositis Center of Excellence (SMCE), Nippon Medical School Hospital, Tokyo 113-8603, Japan

**Keywords:** drug transition study, pulmonary arterial hypertension, pulmonary vascular resistance, selexipag, beraprost

## Abstract

Pulmonary arterial hypertension (PAH) remains a significant challenge in cardiology, necessitating advancements in treatment strategies. This study explores the safety and efficacy of transitioning patients from beraprost to selexipag, a novel selective prostacyclin receptor agonist, within a Japanese cohort. Employing a multicenter, open-label, prospective design, 25 PAH patients inadequately managed on beraprost were switched to selexipag. Key inclusion criteria included ongoing beraprost therapy for ≥3 months, a diagnosis of PAH confirmed by mean pulmonary artery pressure (mPAP) ≥ 25 mmHg, and current treatment with endothelin receptor antagonists and/or phosphodiesterase type 5 inhibitors. Outcomes assessed were changes in hemodynamic parameters (mPAP, cardiac index, pulmonary vascular resistance) and the 6 min walk distance (6-MWD) over 3–6 months. The study found no statistically significant changes in these parameters post-switch. However, a subset of patients, defined as responders, demonstrated improvements in all measured hemodynamic parameters, suggesting a potential benefit in carefully selected patients. The transition was generally well-tolerated with no serious adverse events reported. This investigation underscores the importance of personalized treatment strategies in PAH, highlighting that certain patients may benefit from switching to selexipag, particularly those previously on higher doses of beraprost. Further research is needed to elucidate the predictors of positive response to selexipag and optimize treatment regimens for this complex condition.

## 1. Introduction

Pulmonary arterial hypertension (PAH) is a progressive and life-threatening disorder characterized by elevated pulmonary arterial pressure and pulmonary vascular resistance, leading to right heart failure and premature death [1]. Despite significant advances in the understanding of PAH pathogenesis and the development of targeted therapies, the prognosis for patients with PAH remains poor [2]. Current treatment strategies aim to improve symptoms, exercise capacity, and hemodynamics while delaying clinical worsening and death [3,4].

Pharmacological treatment of PAH typically involves the use of several classes of drugs, including endothelin receptor antagonists (ERAs), phosphodiesterase type 5 inhibitors (PDE5i), and prostacyclin analogs [1]. These agents target different pathways involved in the pathogenesis of PAH, such as the endothelin, nitric oxide, and prostacyclin pathways, respectively [5].

Endothelin-1 (ET-1) is a potent vasoconstrictor and promoter of vascular remodeling, and its levels are elevated in patients with PAH [6]. ERAs, such as bosentan, ambrisentan, and macitentan, block the binding of ET-1 to its receptors, resulting in vasodilation and reduced vascular remodeling [7]. Clinical trials have demonstrated that ERAs improve exercise capacity, hemodynamics, and clinical outcomes in patients with PAH [8,9]. In particular, the SERAPHIN trial showed that macitentan, a novel dual endothelin receptor antagonist, significantly reduced morbidity and mortality in patients with PAH compared to a placebo [10].

The nitric oxide-soluble guanylate cyclase-cyclic guanosine monophosphate (NO-sGC-cGMP) pathway plays a crucial role in regulating pulmonary vascular tone and endothelial function. Impairment of this pathway contributes to the pathogenesis of PAH, and its modulation has emerged as a key therapeutic target [11,12]. Soluble guanylate cyclase stimulators, such as riociguat, have shown promising results in the treatment of PAH by enhancing cGMP production and promoting vasodilation [13].

Prostacyclin analogs have been a mainstay of PAH treatment for decades, with demonstrated benefits in improving hemodynamics, exercise capacity, and survival [2]. Beraprost, an orally active prostacyclin analog, has been used in Japan since the early 2000s for the treatment of PAH [14]. However, its efficacy has been limited by its short half-life and suboptimal pharmacokinetic profile [15].

Selexipag, a novel oral selective prostacyclin receptor agonist, has emerged as a promising therapy for PAH [16]. The GRIPHON trial demonstrated that selexipag significantly reduced the risk of the primary composite endpoint of death or a complication related to PAH compared to placebo.

Given the limitations of beraprost and the potential benefits of selexipag, transitioning patients from beraprost to selexipag may be a viable strategy to optimize treatment outcomes in PAH. However, data on the safety and efficacy of this approach are limited. A recent retrospective study suggested that switching from beraprost to selexipag may be safe and effective in children and young adults with idiopathic and heritable PAH [17]. To our knowledge, no prospective studies have evaluated the outcomes of transitioning from beraprost to selexipag in a multicenter setting.

Therefore, the aim of this study was to investigate the safety and efficacy of switching from beraprost to selexipag in patients with PAH who were inadequately managed on beraprost. We hypothesized that transitioning to selexipag would lead to improvements in hemodynamics and exercise capacity without compromising safety. This study provides valuable insights into the potential role of selexipag in optimizing treatment strategies for PAH and contributes to the growing body of evidence supporting personalized approaches to PAH management.

To our knowledge, this is the first prospective, multicenter study to investigate the safety and efficacy of transitioning PAH patients that are inadequately managed on beraprost to selexipag. By addressing this specific gap in the field, our study provides novel insights into the potential benefits and risks of this treatment strategy in a Japanese PAH cohort, with implications for clinical decision making and future research.

## 2. Results

### 2.1. Patient Characteristics

The baseline characteristics of the study population were as follows: the mean age was 50.2 years (±3.1), with 84% (21 patients) being female. The mean pulmonary artery pressure (PAP) was 38.1 mmHg (±1.7), while the cardiac index was 2.95 L/min/m^2^ (±0.13). Pulmonary vascular resistance (PVR) was 495 dynes·s·cm^−5^ (±40), and the 6 min walk test distance (6MWTD) was 403 m (±29). Patients were distributed across the New York Heart Association (NYHA) functional classes as follows: two patients were in class I, fifteen patients were in class II, and eight patients were in class III. A total of 92% (23 patients) were administered endothelin receptor antagonists (ERA), 84% (21 patients) were given phosphodiesterase type 5 inhibitors (PDE5i) or soluble guanylate cyclase (sGC), and 100% (25 patients) received either ERA, PDE5i, or sGC therapy. The mean follow-up period for the study was 456 days (±78).

The mean (±SE) dose of beraprost before switching was 211 ± 22 mg/day. Following the transition to selexipag, the mean (±SE) dose was 2170 ± 210 mg/day. The majority of patients in our study were receiving concomitant PAH therapies, including ERAs (92%), PDE5i or sGC stimulators (84%), or a combination of these agents (100%). The potential impact of these background therapies on the response to selexipag should be considered when interpreting our findings.

### 2.2. Dosing of Selexipag and Tolerability

The mean (±SE) dose of beraprost before switching was 211 ± 22 mg/day. Following the transition to selexipag, the mean (±SE) dose was 2170 ± 210 mg/day. No serious adverse events related to selexipag were reported during the study period.

### 2.3. Hemodynamic and Exercise Capacity Outcomes

Following the switch from beraprost to selexipag, changes in right heart catheterization parameters and the 6 min walk test distance (6MWTD) were observed. The mean pulmonary artery pressure (PAP) changed from 38.1 ± 1.7 (±SE) mmHg to 37.6 ± 1.9 mmHg, although this change was not statistically significant (*p* = 0.687). The cardiac index demonstrated an increase from 2.95 ± 0.13 (±SE) L/min/m^2^ to 3.17 ± 0.16 L/min/m^2^, but this increase also did not reach statistical significance (*p* = 0.069). Pulmonary vascular resistance (PVR) decreased from 495 ± 40 (±SE) dynes·s·cm^−5^ to 458 ± 43 dynes·s·cm^−5^; however, this change was not statistically significant either (*p* = 0.202). The 6 min walk test distance (6MWTD) showed a slight decrease from 409 ± 29 (±SE) meters to 400 ± 32 m, but this change was not statistically significant (*p* = 0.504).

### 2.4. Responder Analysis

Nine patients (36%) were classified as responders, defined as those who experienced improvements in all three hemodynamic parameters (mPAP, CI, and PVR) after switching to selexipag. The responders had a higher tendency of mean dose of beraprost at baseline compared to non-responders (233 ± 38 vs. 198 ± 27 μg/day, *p* = 0.23). Additionally, responders tolerated significantly higher mean dose of selexipag at the end of the titration period (2755 ± 235 vs. 1900 ± 249 μg/day, *p* < 0.016).

To further characterize the responder and non-responder groups, we analyzed the PAH etiology, disease duration, and other baseline characteristics between the two groups. However, due to the small sample size, no statistically significant differences were observed in these parameters.

In the non-responder group, there were fourteen females (66.67%) and two males (50%), while in the responder group, there were seven females (33.33%) and two males (50%). The total number of patients included twenty-one females and four males.

Regarding age categories, the non-responder group had one patient under 30 years old (50%), one patient aged 30–39 (25%), three patients aged 40–49 (60%), and eleven patients aged 50 or older (78.57%). In the responder group, there was one patient under 30 years old (50%), three patients aged 30–39 (75%), two patients aged 40–49 (40%), and three patients aged 50 or older (21.43%). The total number of patients was two under 30 years old, four aged 30–39, 5 aged 40–49, and fourteen aged 50 or older.

**Figure 1:**
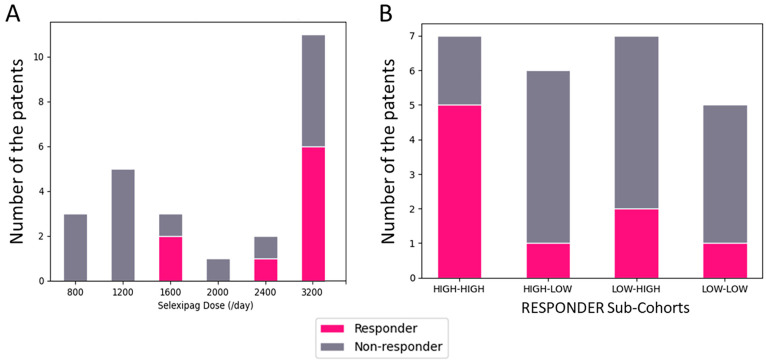
(**A**). Distribution of the cohorts of responders and non-responders, as determined by the daily dose of selexipag. Dose units are in μg/day. (**B**). Responder distribution, as determined by the pre-dose of selexipag and final dose of beraprost is depicted. Sub-cohort of higher-dose of beraprost was defined as participants receiving ≥240 μg/day pre-switch, and the sub-cohort of higher-dose selexipag was defined as participants continuing to tolerate ≥2000 μg/day.

When comparing the nine patients in the responder group and the sixteen patients in the non-responder group, it was observed that the non-responder group tended to remain on a lower dose of selexipag after switching, as shown in Figure 1.

## 3. Discussion

This prospective, multicenter study investigated the safety and efficacy of switching from beraprost to selexipag in patients with PAH who were inadequately managed on beraprost. To our knowledge, this is the first study to evaluate this transition in a prospective, multicenter setting. The main findings of our study were: (1) switching from beraprost to selexipag was safe and well-tolerated, with no serious adverse events related to selexipag; (2) although there were no significant overall improvements in hemodynamics or exercise capacity, a subgroup of patients who experienced significant improvements in all hemodynamic parameters were characterized by having a tolerance to higher doses of beraprost at baseline.

The lack of significant overall improvements in hemodynamics and exercise capacity in our study may be related to several factors. First, the study population had relatively stable disease, with a mean baseline PVR of 495 dyn·s·cm^−5^ and a mean 6-MWD of 403 m. Second, the mean dose of selexipag achieved in our study (2170 μg/day) was lower than the maximum dose used in the GRIPHON study (3200 μg/day), which may have limited the potential for improvement [16]. Finally, the duration of follow-up in our study (mean of 456 days) may have been too long to detect beneficial changes in hemodynamics and exercise capacity.

Despite these limitations, our study identified a subgroup of patients who experienced significant improvements in all measured parameters after switching to selexipag. These responders were characterized by receiving higher doses of beraprost at baseline and higher doses of selexipag at the end of titration. This finding suggests that patients who have been on higher doses of beraprost and demonstrate a high tolerance for selexipag may be more likely to benefit from this transition. This is consistent with previous studies showing a dose–response relationship for the efficacy of selexipag [18].

The predictive value of beraprost dose at baseline for response to selexipag is a novel finding of our study. This may reflect a subset of patients who are more sensitive to prostacyclin pathway modulation and are, therefore, more likely to respond to selexipag. Alternatively, it may suggest that patients who have been on higher doses of beraprost have more severe disease and thus more room for improvement with selexipag. Further studies are needed to clarify the mechanisms underlying this association and to validate the use of beraprost dose as a predictor of response to selexipag.

Our study has several strengths, including its prospective, multicenter design and the use of rigorous inclusion criteria and standardized dosing protocols. However, there are also limitations to consider. First, the sample size was relatively small, which may have limited our power to detect significant overall differences in hemodynamics and exercise capacity. Second, the study was open-label and did not include a control group, which may have introduced bias. Third, the duration of the follow-up was relatively short, and longer-term studies are needed to evaluate the sustained efficacy and safety of selexipag in this population.

One limitation of our study is the relatively small sample size of 25 patients, which may have limited our power to detect significant overall differences in hemodynamics and exercise capacity. Larger studies are needed to provide more robust conclusions regarding the efficacy of switching from beraprost to selexipag in PAH patients. Another limitation of our study is the relatively short mean follow-up duration of 456 days, which may not have been long enough to fully evaluate the long-term efficacy and safety of switching from beraprost to selexipag. Extended follow-up data would be valuable to assess the sustained benefits and potential long-term adverse effects of this treatment strategy. 

Additionally, the open-label design of our study and the lack of a control group of patients maintained on beraprost introduce potential bias and limit our ability to directly compare outcomes between the two treatment strategies. Including a control arm would have strengthened our findings by allowing a more robust assessment of the efficacy of switching from beraprost to selexipag compared to continuing beraprost therapy. The unbalanced sample composition, with a higher proportion of females and an uneven distribution across NYHA functional classes, may have influenced the observed treatment responses. Evidence suggests that gender and NYHA functional class can impact PAH outcomes [19]. Future studies with larger, more balanced cohorts are needed to investigate the potential confounding effects of these factors on the efficacy and safety of transitioning from beraprost to selexipag. 

While our study identified a potential responder phenotype characterized by higher beraprost and selexipag dosing, more detailed analyses comparing factors such as PAH etiology, disease duration, and other baseline characteristics between responders and non-responders were limited by the small sample size. Future studies with larger cohorts should aim to better characterize the factors that may predict a favorable response to selexipag after switching from beraprost. As our study was conducted in a Japanese PAH patient population, the generalizability of our findings to other populations may be limited. Differences in genetic background, lifestyle factors, and healthcare systems could potentially influence treatment responses and outcomes. Confirming our findings in diverse patient cohorts would increase the external validity of the study and provide a more comprehensive understanding of the efficacy and safety of switching from beraprost to selexipag in PAH patients worldwide.

## 4. Materials and Methods

### 4.1. Study Design

The study was designed as a multicenter, prospective, observational study, with the aim of enrolling 25 participants and collecting follow-up data from enrollment (observation start) to follow-up. Right heart catheterization (RHC), NYHA classification, exercise capacity, and PA/PAH-specific treatment data were collected at 3–6 months. Participants were followed from the time of switching from beraprost sodium to selexipag until RHC at 3–6 months after the switch. No restrictions were placed on appropriate supportive therapies. Additionally, information on the switching dose of selexipag from beraprost was collected at follow-up. Regarding dose setting, the maximum tolerated dose was determined by the physician based on patient tolerability.

The primary endpoint was pulmonary vascular resistance (PVR), assessed at enrollment and follow-up (3–6 months later).

Beraprost sodium dosage was not specifically regulated at enrollment. It was assumed that the maximum tolerated dose would be used, and both immediate-release and sustained-release formulations were treated as equivalent if the dosages were the same. Regarding concomitant medications rules, the following PAH medications were prohibited for use during the study: epoprostenol, treprostinil, and iloprost. There were no specific restrictions on the use of the following concomitant medications, but their dosages were stable during the study: bosentan, ambrisentan, macitentan, sildenafil, tadalafil, and riociguat. 

This study was conducted in accordance with the amended Declaration of Helsinki. The study was approved by an institutional review board and written informed consent was obtained from all participants. Given the exploratory nature of this study, no formal sample size calculation was performed. 

### 4.2. Inclusion Criteria

Participants were required to meet the following criteria: (a) be 18 years or older at the time of consent, (b) be diagnosed with PAH (Nice classification Group 1), (c) provide written informed consent after receiving sufficient explanation and understanding of the study, (d) be currently taking endothelin receptor antagonists (bosentan, ambrisentan, macitentan) and/or PDE-5 inhibitors/soluble GC stimulants (sildenafil, tadalafil, riociguat) or not taking any PAH medication other than beraprost sodium, (e) have a WHO functional class I to III at enrollment, (f) have a 6 min walk distance (6MWD) between 150 and 450 m within 30 days of starting selexipag, (g) have a mean pulmonary artery pressure (mPAP) ≥ 25 mmHg within 30 days of starting selexipag, and (h) have been continuously taking a stable dose of beraprost sodium for at least 3 months before switching.

### 4.3. Data Collection

Data collection for this study was based on the Japanese Association of Pulmonary Hypertension Registry (JAPHR) input items and divided into two stages: baseline and follow-up. The data were recorded in the Japan PH Registry’s electronic data capture system [10].

### 4.4. Statistical Analysis

We report the characteristics of the patients in means and standard deviations for the continuous variables and in counts and percentages for categorical variables. We also evaluated the change in mean pulmonary artery pressure (PAP), pulmonary vascular resistance (PVR), and cardiac index by subtracting the values at baseline from those at the follow-up assessment. We defined the patients with improvement in the above three values (change in the values as below 0 for mean PAP, PVR, and above 0 for CI) and in the classification of NYHA as the improved group, and the others as non-improvement group. We compared the characteristics of these two groups for their sex, age, baseline NYHA, and baseline beraprost dose as well as the maximum selexipag dose post medication switch.

Analyses were performed using SAS version 9.4 (Cary, NC, USA). Data regarding the dose of selexipag at the point of switching from beraprost were collected. Data collection was divided into baseline and follow-up stages. A paired sample *t*-test was used to investigate the differences between paired samples. We conducted a chi-square test of independence for categorical data. In both tests, the significance level (α) was set at 0.05. A *p* value less than this threshold was considered statistically significant.

## 5. Conclusions

In conclusion, this prospective, multicenter study demonstrated that switching from beraprost to selexipag was safe and well-tolerated in a small cohort of Japanese PAH patients who were inadequately managed on beraprost. Although there were no statistically significant improvements in hemodynamics or exercise capacity in the overall study population, a subgroup of patients who were on higher doses of beraprost at baseline and tolerated higher doses of selexipag experienced improvements in all measured parameters. However, given the small sample size and exploratory nature of our study, these findings should be interpreted with caution. A larger, appropriately powered randomized controlled trial is needed to confirm the efficacy and safety of transitioning from beraprost to selexipag in PAH patients. Our study provides preliminary insights into the potential role of selexipag in optimizing treatment strategies for PAH and highlights the need for further research to guide personalized treatment approaches in this complex condition.

## Data Availability

The data in this study is available from the corresponding author upon reasonable request.
